# Utilizing asynchronous email interviews for health research: overview of benefits and drawbacks

**DOI:** 10.1186/s13104-021-05547-2

**Published:** 2021-04-20

**Authors:** Michelle Amri, Christina Angelakis, Dilani Logan

**Affiliations:** 1 Dalla Lana School of Public Health, University of Toronto, 155 College Street, Toronto, ON M5T 1P8 Canada; 2 Takemi Program in International Health, Harvard School of Public Health, Harvard University, 665 Huntington Avenue, Bldg. 1, Room 1210, Boston, MA 02115-6021 USA; 3 School of Public Health and Social Policy, University of Victoria, 3800 Finnerty Road, Victoria, BC V8P 5C2 Canada; 4 University of Western Ontario, 1151 Richmond St, London, Ontario N6A 3K7 Canada; 5 American Institutes for Research, 2800 Campus Drive, Suite 200, San Mateo, CA 94403 USA

**Keywords:** Asynchronous electronic interviews, Qualitative method, Research method, Global health research, Health research, Qualitative research, Email interviews, E-interviews, Asynchronous computer-mediated communication

## Abstract

**Objective:**

Through collating observations from various studies and complementing these findings with one author’s study, a detailed overview of the benefits and drawbacks of asynchronous email interviewing is provided. Through this overview, it is evident there is great potential for asynchronous email interviews in the broad field of health, particularly for studies drawing on expertise from participants in academia or professional settings, those across varied geographical settings (i.e. potential for global public health research), and/or in circumstances when face-to-face interactions are not possible (e.g. COVID-19).

**Results:**

Benefits of asynchronous email interviewing and additional considerations for researchers are discussed around: (i) access transcending geographic location and during restricted face-to-face communications; (ii) feasibility and cost; (iii) sampling and inclusion of diverse participants; (iv) facilitating snowball sampling and increased transparency; (v) data collection with working professionals; (vi) anonymity; (vii) verification of participants; (viii) data quality and enhanced data accuracy; and (ix) overcoming language barriers. Similarly, potential drawbacks of asynchronous email interviews are also discussed with suggested remedies, which centre around: (i) time; (ii) participant verification and confidentiality; (iii) technology and sampling concerns; (iv) data quality and availability; and (v) need for enhanced clarity and precision.

## Introduction

‘Email interviewing’^1^ is a relatively new research method, which offers great potential for qualitative researchers [1]. There are two main categories of email interviews: asynchronous and synchronous [2]. In asynchronous interviews, respondents can receive questions by email or video, which they can reply to at their convenience. In synchronous interviews, both the interviewer and interviewee are online at the same time and questions are posed sequentially in real-time [2]. Asynchronous email interviews, can take place over a variety of time intervals (i.e. hours, days, weeks or months), and therefore offer more flexibility than synchronous email approaches [3]. It is for this reason and others, that we explore asynchronous email interviews in this article, providing an overview of the benefits and drawbacks. Our insights are based on an analysis of the existing literature and personal reflections from one of the author’s prior usage of this method.

## Methods

### Narrative review and gleanings from tobacco policy research project

With the current climate of COVID-19 pushing many data collection efforts online, we undertook this narrative review of the relatively new method of ‘email interviewing’, with particular attention paid to asynchronous email interviews, to assist researchers with their online data collection efforts. As such, our narrative review focuses on collating an overview of the benefits and drawbacks of asynchronous email interviewing. We also reflected upon the unique insights elicited by one of our author’s prior experiences utilizing asynchronous email interviews. The author conducted a study canvasing public health practitioners’ perspectives on tobacco policy tools, using email to both recruit participants and conduct asynchronous email interviews [4]. These insights have been embedded into the results.

## Results

### Benefits of asynchronous email interviewing

#### Access transcending geographic location and during restricted face-to-face communications

Asynchronous email interviews enable data collection from beyond the researcher’s physical location. This ability to transcend geography is particularly useful during the COVID-19 pandemic, as asynchronous email interviewing allows continued engagement with research and provides participants with time to collect and articulate their thoughts, while concurrently ensuring the safety of both participants and researchers.

#### Feasibility and cost

Asynchronous email interviews can reduce the time required to conduct a research study while still generating in-depth data [5]. The portable nature of asynchronous email interviewing also facilitates response rates, as participants can answer research questions from locations with an internet signal [3]. Similarly, saving scheduling and travel time, and subsequently reducing research costs [6], such as reduced transportation fees [5] and transcription services which become unnecessary [7]. It also allows researchers to conduct multiple interviews simultaneously [1], reducing work hours and/or affording time to complete other tasks and improving productivity [5]. The culmination of cost and time savings can result in larger resource allocations for analysis tasks and/or reap benefits for researchers with limited budgets [3].

#### Sampling and inclusion of diverse participants

For participants of lower of socioeconomic status, access to strong, unobstructed internet connections can at times be a limitation to participating in synchronous electronic interviews. However, asynchronous email interviewing mitigates issues of internet drop-off and poor connection, by providing participants with the ability to formulate and send responses in spite of connection challenges. Moreover, this method can help prevent the exclusion of participants from lower socioeconomic status due to technological limitations. Even if participants cannot afford an internet connection at home, they can either bring their electronic device to community locations or use public computers (e.g. public libraries).

The internet’s global reach also allows researchers to transverse geographic regions when recruiting, resulting in potentially larger sample sizes [5] and increased access to varied opinions and points of view [6]. Since both parties are not required to be active online at the same time, this also eliminates the barrier of time zone variabilities [6]. Email interviewing also enables involvement from participants who would otherwise face limitations to in-person interviewing, including persons with disabilities or those who are location-bound [8]—with asynchronous email interviewing being particularly mindful of constraints placed on individuals. Therefore, increased sampling and inclusion of participants reduces accessibility barriers, broadens and diversifies sample sizes, and can increase the generalizability of study results [9].

#### Facilitating snowball sampling and increased transparency

Snowball sampling involves one participant referring another potential participant. Asynchronous email interviewing allows the first participant to easily forward detailed requests, and additional participants to freely partake in the study, given the lack of geographic, time, and accessibility barriers typically faced in face-to-face interviews.

In the tobacco policy project, participants volunteered to forward the letter of information and consent form to other potential participants. While it is difficult to discern whether this may have also occurred using other qualitative methodologies, we speculate that having the information clearly presented with an option to easily forward at the touch of a button without having to disclose the potential participants’ names to the researcher, made it substantially easier to pass along this information.

#### Data collection with working professionals

In the study of public health practitioners, these individuals had professional obligations which likely entails availability constraints. Use of asynchronous email interviews was particularly beneficial for engaging this population, as they were free to respond at a time that was most convenient to them. Moreover, the professionals in the study were contacted due to their role at a particular organization or institution. If participants wanted to ensure they were accurately representing their role or organization, the asynchronous email interview method allowed them to carefully curate and edit their typed responses before submission to the researcher. While this may result in less spontaneous answers that occur during face-to-face interactions, it poses an opportunity to garner information from professionals who may have otherwise not obliged to interview requests due to their institutional affiliations.

#### Anonymity

Allowing individuals to express their responses regarding a sensitive topic in a written form can also take on a therapeutic role [3] and may promote feelings of safety when disclosing sensitive information [3]. Not physically being present in front of a researcher increases feelings of anonymity and prevents participants from feeling self-conscious of their physical appearance [7]. For especially sensitive topics, researchers should ensure they are properly redirecting participants to supports [5].

#### Verification of participants

In this study of public health practitioners, participants were emailed at their institutional email address, aiding with participant verification (and institutional websites can further verify biographies).

#### Data quality and enhanced data accuracy

Time-pressure can result in inconsistent responses, spelling errors, and miscommunication. Therefore, by not requiring an instantaneous response, researchers alleviate sentiments of anxiety and stress amongst participants who struggle with expressing their thoughts instantaneously [5]. By giving participants sufficient time to reflect on the question before formulating a cohesive answer [7], the accuracy of email transcripts may be increased, as participants have time to proofread their responses [5]. Increased response time also provides researchers with the opportunity to reflect more on the information already stated and pose appropriate follow-up questions [9]. Additionally, verbal fillers such as “um”, “uh”, and “like” may not be typed out by respondents, potentially increasing the overall quality of the data [8].

#### Overcoming language barriers

Using computer applications to translate participant responses can mitigate traditional language barriers posed by face-to-face interviewing and eliminate the need for human translators and potentially scheduling challenges. Future studies can remedy language barriers posed by asynchronous email interviews by generating letters of information, consent forms, and interview questions in languages that are used by the target participants.

### Drawbacks of asynchronous email interviewing

#### Time

If a predetermined window for response submission is not established, participants may take months to reply [6, 7]. Moreover, responses that are sent sporadically interrupt the flow of discussion and delay data collection [6]. This can be particularly true for sensitive subject matter, where respondents may feel emotionally distressed [5]. It is also important to consider the appropriateness of asynchronous versus synchronous interviewing methods for distressing topics.

#### Participant verification and confidentiality

Email interviews require researchers to exhibit a high degree of diligence and carefully vet each participant [5]. Participants may also have reservations about their privacy [1]. For instance, in the tobacco policy study, one participant inquired about anonymity and opted out. As is common across all forms of data collection, it is important to communicate how precautions are being taken to protect participant privacy (e.g. using a secure platform).

Researchers need to be cautious when constructing emails to avoid forwarding confidential details [8] or mixing up narratives when responding to participants concurrently [3]. This can be prevented through increased organization and scheduling. Colour coding email correspondences and storing conversations from different participants in separate inbox folders can help prevent these occurrences. Similarly, researchers may use a secure emailing portal to manage confidential correspondence and prevent susceptibility to virtual hacking or phishing scams.

#### Technology and sampling concerns

While technology has afforded novel interviewing methods, it simultaneously poses challenges. For instance, poor internet connectivity may discourage individuals from participating or delay their responses, and system crashes can result in the elimination of data [8]. Many potential participants may also have inactive email accounts or avoid reading research invitations, straining recruitment efforts [5]. However, these issues are not unique to email interviewing and can be overcome, such as through building connections with gatekeepers at participants’ institutions, using recruitment tools, and gaining a thorough understanding of the target population during the study planning stages [10]. Modifying email subject lines and directly addressing emails to potential participants may also mitigate traditional issues.

Furthermore, research participants may be from higher socioeconomic strata, limiting study generalizability and resulting in sampling bias [5]. Similarly, technological literacy may hinder response collection, such as from those with limited rapid typing skills to provide full answers [8] and those who may not be as comfortable using computers and the internet (e.g. some aging individuals) [9]. However, given the decreased pressure to respond quickly, many of the aforementioned technological issues may be overcome.

While the feeling of being anonymous may yield benefits, as discussed above, it is also important to consider how a feeling of lack of anonymity may result in sampling bias. For example, participants who opt out due to feeling uncomfortable with the asynchronous electronic interview method will result in sampling bias and potentially skew results. It is therefore crucial for the researcher to take actions to mitigate any feelings of lack of anonymity and consider any implications on sampling bias while undertaking recruitment.

#### Data quality and availability

Asynchronous email interviewing fails to account for non-verbal and paralinguistic cues [5]. While the exclusion of such cues may yield benefits, as discussed above with respect to filler words, this is not the case for all studies, as a mutual failure to build human connection via expressions and non-verbal cues may limit participant willingness to build trust with the interviewer and disclose sensitive details [6]. As such, it is the responsibility of the researcher to weigh the benefits and drawbacks of the omittance of such cues in determining the appropriateness of the asynchronous electronic interview method.

Respondents may also perceive the lack of face-to-face meetings as researcher laziness and reciprocate with decreased effort in their responses (e.g. brevity), possibly resulting in numerous time-consuming follow-ups [5]. Researchers should ensure questions are self-explanatory, easy to interpret, and pay attention to email tone and word-use to convey warmth.

The use of emoticons has been suggested as an alternative to visual cues and can convey warmth [11]. While verbal intonations and emphasis can also be expressed through repetitive characters, for example “weeeelllll, meeee???, ouuuuu” [9]; these intonations should not be used by researchers to maintain professionalism.

#### Need for enhanced clarity and precision

In the tobacco policy study, one respondent indicated that “there are concerns and implications due to the regressive nature of tobacco taxation increases” [4]. It was unclear what “regressive nature” meant when writing up results, which led the researcher to draw on contextual clues to inform her understanding [4]. Researchers must use clear and precise language in correspondence, and ensure key terms are defined at the outset. Similarly, it is recommended that researchers review data collected from participants as soon as it is received and with enhanced attention to detail, as it will allow researchers to immediately ask follow-up questions.

## Conclusions

The above findings yield considerations for future studies seeking to employ the underutilized method of asynchronous email interviews, particularly pertinent during COVID-19. We highly advise all researchers who are assessing the utility of this method to carefully consider this compilation of benefits and drawbacks and reflect on the appropriateness for the aim of their study and type of participants being sought. It is important to note that while asynchronous email interviews may be a preferred option for one study, this may not be the case for another study.

Through the inherent nature of asynchronous email interviews being able to collate data from across the world, we hope this method will also aid in elevating the voices from the global south and those of women—which is sorely needed in global health leadership (12)—whether through better understanding unique perspectives or assisting in the building of cross-country research partnerships.

## Limitation

While this article collates both benefits and potential drawbacks of asynchronous email interviewing through drawing on various studies and observations from one author’s study, this list is not exhaustive and new considerations may emerge through undertaking this method.

## Data Availability

Data sharing is not applicable to this article as no datasets were generated or analysed during the current study.

## Footnotes

1 Also known as ‘e-interviewing’, ‘electronic interviewing’, and ‘asynchronous computer-mediated communication’.
